# Treadmill training induces sex-dependent changes in hippocampal epigenetic patterns and plaque-associated microglial morphology in aged TgF344 rats

**DOI:** 10.3389/fnins.2026.1805957

**Published:** 2026-05-05

**Authors:** Adam J. Schuller, Megan R. Hager, Aidan M. Briggs, Savannah M. Rocha, Omar A. Yanouri, Emma J. Smith, Stephanie E. Hall, Luke B. Montrose, Ronald B. Tjalkens

**Affiliations:** 1 Department of Environmental and Radiologic Health Sciences, Colorado State University, Fort Collins, CO, United States; 2 Department of Anatomy and Physiology, Kansas State University, Manhattan, KS, United States

**Keywords:** Alzheimer’s disease, DNA methylation, epigenetics, neuroinflammation, physical exercise

## Abstract

Alzheimer’s disease (AD) is the most prevalent neurodegenerative disorder world-wide, characterized by progressive neuroinflammation, aberrant protein accumulation, and neuronal loss associated with cognitive decline. Although our understanding of the molecular mechanisms underlying AD pathogenesis has greatly increased in recent years, there remain limited treatment strategies and no cures for this disorder. Because of this, efforts have shifted toward identifying modifiable lifestyle factors which may decrease risk of onset or slow AD progression. One such approach which has shown promise in modulating the disease course is physical exercise. However, sex-specific effects of implementing such activity strategies in aged individuals after the onset of disease are less well studied. We sought to address this knowledge gap by characterizing hippocampal histopathology and DNA modification profiles of aged TgF344-AD rats following progressive treadmill-training. Reduced-representation bisulfite sequencing indicated 94 genes associated with differentially modified cytosines (DMCs) in exercised females (239 differentially modified regions, 54.6% hypermodified) and 87 DMC-associated genes in exercised males (216 differentially modified regions, 50.4% hypermodified) with unique functional enrichment for overrepresented pathways and protein interactions relevant to glial activation and synaptic plasticity. Using quantitative high-throughput slide scanning fluorescence microscopy we additionally examined this brain region for AD-relevant changes including neuronal and microglial density, microglial morphology, and accumulation of pathologic protein. This analysis revealed female-specific reductions in NeuN^+^ and Iba1^+^ cells in treadmill-trained animals, as well as sex- and exercise-dependent changes in plaque-associated microglial reactivity state. Together, these findings reveal that age of onset, biologic sex, and duration of physical exertion may be important factors in modulating the pathologic progression of AD.

## 1 Introduction

Alzheimer’s disease (AD) is the most common cause of dementia worldwide (Anonymous, 2024). This progressive, age-related neurodegenerative disorder can be separated into several subtypes, the most common being late onset AD (LOAD), which remains largely idiopathic with respect to etiology (Rabinovici, 2019). Accompanying this disease is a devastating clinical phenotype of executive dysfunction and decrements in learning and memory that worsen over time. The single greatest risk factor for LOAD remains advanced age, with the majority of individuals being diagnosed after the age of 65 (Rabinovici, 2019). Genetic mutations and environmental factors that correlate with disease incidence have been identified, but none strongly predict disease risk across all populations (Finch and Kulminski, 2019). Current therapeutic strategies have been primarily aimed at reducing the pathologic burden of senile plaques, comprised of misfolded amyloid beta (Aß), and neurofibrillary tangles, characterized by hyperphosphorylated microtubule associated protein tau (MAPT) (Pardo-Moreno et al., 2022). Aggregation of these proteins tends to arise in the hippocampus and entorhinal cortex and is accompanied by inflammatory activation of microglia and astrocytes as well as an eventual loss of neurons.

Recent reports have demonstrated that changes in glial cell morphology and patterns of gene expression associated with neuroinflammation precede overt neurodegeneration (Park and Barrett, 2020; Rohden et al., 2025). Microglia function as resident immune cells in the CNS and play a critical role in a variety of essential biological processes, such as synaptic maintenance and pruning, regulation of myelination, phagocytosis, and inflammatory responses (Li and Barres, 2018; McNamara et al., 2023). Reactive states of microglia are highly dynamic and can have both neuroprotective and neurotoxic effects. Microglia contribute to homeostasis by surveying the brain parenchyma aiding in autophagy of misfolded proteins, synaptic maintenance, and identification of pathogens (Li and Barres, 2018). Additionally, reactive microglia communicate with other immune cell types via inflammatory signaling that can contribute to neuronal injury (Hanisch and Kettenmann, 2007). In neurodegenerative disease, chronically activated microglia may fail to endocytose pathologic protein aggregates, thereby amplifying neuronal dysfunction (Cai et al., 2022). This two-fold role of microglia in the pathogenesis and progression of AD is one of the challenges in identifying molecular targets for therapy.

Modifiable lifestyle factors, such as physical activity throughout the life course, are posited to protect against AD by increasing neuroprotective mechanisms such as neurogenesis and synaptic plasticity, while decreasing neuroinflammation (Tari et al., 2025). Physical exercise has shown a demonstrated benefit in both human studies and in animal models of AD (De la Rosa et al., 2020). Novel evidence suggests that benefit may be due to reductions in microglial proliferation and inflammation (Strohm and Majewska, 2024). However, the molecular basis for the effects of physical exercise on pathogenic mechanisms in AD is not fully understood, especially in the context of aging and pre-existing disease. Some studies have explored the effects of exercise in disease models that do not recapitulate all of the hallmark pathologic features of AD (Belarbi et al., 2011; Gratuze et al., 2017). Other groups have performed studies in disease models without examining the effects of biologic sex, despite known sex differences in physiologic and cellular responses to exercise (Li et al., 2025; Liu et al., 2020). These challenges are further compounded by reports that intense exercise in transgenic models of neurodegenerative disease can induce mitochondrial stress sufficient to exacerbate neuronal injury in genetically susceptible animals (Sliter et al., 2018).

Insight into mechanisms by which the age- and sex-dependent effects of exercise can be separated may be provided by more thorough characterization of epigenetic profiles in these contexts. Alterations in epigenetic patterns, which are capable of affecting gene expression without directly altering the DNA sequence itself, have been reported in peripheral blood mononuclear cells (PBMCs), as well as in the hippocampus, following physical activity (Zheng et al., 2025; Barron-Cabrera et al., 2019). The most well-characterized epigenetic mechanism is DNA modification, classically described as methylation at the 5-carbon of a cytosine in a cytosine-phosphate-guanine (CpG) dinucleotide. DNA modifications can also occur in non-CpG contexts or via other stable modifications of cytosines, and these alternate marks have been shown to be uniquely enriched in the brain parenchyma (Jang et al., 2017). DNA modification is known to be susceptible to environmental exposures as well as lifestyle modifications and changes in these patterns have been implicated both as mechanistic contributors to the progression of AD and as potential biomarkers of the disease process (Behl et al., 2024; Sharma et al., 2020). Here, we sought to address conflicting findings in our understanding of exercise-induced effects in AD by implementing a progressive treadmill training regimen in aged TgF344-AD rats, a transgenic model recapitulating a number of the pathologic features of the disease. We subsequently analyzed hippocampal tissue from these animals against control counterparts, stratifying by sex, for changes in DNA modification patterns, histologic profiles of neurons and glia, as well as the extent of pathologic protein burden.

## 2 Methods

### 2.1 Animal care and use statement

All experiments utilizing animal models were conducted in accordance with NIH guidelines and protocols which were reviewed and approved by the Institutional Animal Care and Use Committee (IACUC) at Boise State University. Twelve-month-old male and female TgF344-AD rats, bred on Fischer 344 background, were generated via breeding stock obtained from Dr. Terrence Town at the University of Southern California (Los Angeles, CA, USA). This transgenic strain has been extensively characterized in the literature previously, including time course of AD phenotype onset and progression, as well as noted sex differences between males and females which reflect the human presentation of the disease (Cohen et al., 2013). All rats (*n* = 6 animals/sex/group) were housed in clear plastic cages in groups of 2 on a 12:12 light:dark cycle with ad libitum food and water. Starting at 12 months of age, a progressive 6-month exercise regimen was implemented for 5 days/week, as previously described (Lopez et al., 2024). Briefly, 1 week prior to treadmill intervention, all animals were acclimatized to the treadmill. On day one of acclimatization, all animals were placed in an individual lane for 5 min. On days 2–5, all animals ran on the treadmill for 5 min at 15 m/min on a 10° incline. During the acclimatization, any animal that refused compliance was removed from the treadmill and placed in the control group, which only accounted for <5% of the study population. Animals were then randomly assigned to control or treadmill-trained groups. Training was completed during the dark cycle and began at 15 m/min on a 10° incline for 15 min during week one, and progressed by 5 min/week to a maximum of 15 m/min for 60 min. At 16 months of age, the duration was decreased to 40 min. The treadmill contained a shock grid that was used at a low intensity (0.2 mA) to motivate animals. If an animal experienced a shock three times during a session, they were removed for a 5-min rest before being placed back onto the treadmill. If an animal had to rest twice during a given training period, they were excluded from the remainder of that session. If more than one of the five animals were removed for the day, the intensity was deemed too great and the incline was changed to 0°. All treadmill training occurred in the animal holding room to ensure sedentary animals were exposed to the same environments. At 18 months of age, all rats were sacrificed and whole brains were removed and sagittally hemisected. One hemisphere was subsequently post-fixed in 10% normal buffered formalin (NBF) for histopathologic assessment while the other hemisphere was flash frozen in liquid nitrogen and stored at −80 °C prior to epigenetic profiling.

### 2.2 DNA extraction and reduced-representation bisulfite sequencing (RRBS)

In order to investigate DNA modification patterns, approximately 15 mg of micro-dissected hippocampal tissue (*n* = 4 animals/sex/group) was collected from the flash frozen brain hemisphere and subjected to DNA extraction via a commercially available kit (DNeasy; Qiagen; Hilden, Germany) following the manufacturer’s protocol for tissue samples. Extracted DNA samples were checked for concentration and purity by NanoDrop spectrophotometry and stored at −20 °C until shipment to Novogene (Beijing, China). DNA concentration and quality were confirmed via Qubit and agarose gel electrophoresis. Genomic DNA was spiked with lambda DNA prior to digestion with restriction enzyme MspI, which has been shown to function in a methylation-insensitive manner (Pornthanakasem et al., 2008). Subsequently, digests were repaired by A-tailing and sequencing adapters were ligated (5′ Adapter: 5′-AGATCGGAAGAGCGTCGTGTAGGGAAAGAGTGT-3′ and 3′ Adapter: 5′-GATCGGAAGAGCACACGTCTGAACTCCAGTCAC-3′). Size-selected fragments (40 to 220 bp) were then bisulfite treated using a commercially available kit (EZ DNA Methylation Gold; Zymo Research; Irvine, CA, USA). Bisulfite converted fragments were PCR amplified and then checked for size and concentration with Agilent 2,100 bioanalyzer and Qubit fluorometer, respectively, before being sequenced via paired-end 150 bp reads (PE150) approach on the Illumina HiSeq platform.

### 2.3 RRBS analysis pipeline

Raw reads (>30 million/sample) were first processed using FastQC (fastqc_v0.11.5) to generate quality metrics across the sequencing dataset. Trimmomatic (trimmomatic_v0.36) was then utilized to clean reads, eliminating adapter contaminated or low-quality reads, with the following parameters: SLIDINGWINDOW:4:15; LEADING:3; TRAILING:3; ILLUMINACLIP:adapter.fa:2:30:10; MINLEN:36. Bismark (bismark_v0.16.3) was then used to align cleaned reads to the rat reference genome using the following parameters: score_min L, 0, −0.2, −X 700; dovetail. Aligned reads were directionally indexed using bowtie2 (bowtie2_v2.2.5). Methylation extractor (bismark_methylation_extractor; no_overlap) results were transformed into bigwig format and visualized in IGV browser to calculate bisulfite conversion efficiency (reported in Supplementary material). Differentially modified regions (DMRs) were identified using the R package DSS (DSS_v2.12.0) with the following parameters: delta: 0; adjusted *p*-value threshold: 1 × 10^−5^; minimum length: 50 bp; minimum CpGs: 3, as initially published (Schuller et al., 2021). Briefly, DMRs were annotated to genes in two categories: gene bodies (from TSS to TES) or promoter regions (2 kb upstream of TSS). CpG islands were identified using cpgIalsndExt and repeats were identified using RepeatMasker. Gene Ontology (GO) Biological Process (BP) pathways overrepresentation analysis was conducted on lists compiled from genes annotated to DMRs as described above using clusterProfiler (v4.2.2) with enrichGO function. Statistically significant GO BP terms were determined based on the default Benjamini-Hochberg (BH) procedure with a cutoff score of FDR < 0.05. DMR-annotated genes were further assessed for functional enrichment via the construction of protein–protein interaction (PPI) networks using the STRING database plug-in for Cytoscape (v3.10.3), including known and predicted interactions. The Fruchterman–Reingold force-directed layout was applied via Gephi (v0.10.1) to create PPI network maps.

### 2.4 Multi-plex immunofluorescence staining

Fixed hemispheres were blocked coronally and sectioned using a freezing-sliding microtome at 50 μm intervals throughout the rostro-caudal extent of the hippocampus. Sections were stored in PBS with sodium azide at 4 °C until staining. Immunofluorescent labeling was performed according to the following protocol: antigen retrieval for 30 min at 95 °C in 0.01 M sodium citrate, three 10-min washes at room temperature (RT) in 0.05 M TBS, blocking for 1 h at RT in 0.2% triton-X + 2% donkey serum in 0.05 M TBS, three 10-min washes at RT in 0.05 M TBS, primary antibody incubation for 1 h at 37 °C in 0.05 M TBS, four 10-min washes at RT in 0.05 M TBS, secondary antibody incubation for 30 min at RT in 0.05 M TBS, three 10-min washes at RT in 0.05 M TBS, counterstaining with Hoechst 33342 (1:5000) for 3 min at RT in 0.05 M TBS, four 10-min washes at RT in 0.05 M TBS. Stained sections were then mounted onto charged glass slides using Prolong Gold antifade hard set mounting medium and #1.5 glass coverslips. All slides were stored in the dark at 4 °C until imaging. A full list of antibodies and their dilutions can be found in Tables 1 and 2.

**Table 1:** Primary antibody concentrations and associated product information.

Brand	Product #	Host	Antigen	Lot #	Dilution
Invitrogen	44–136	Rabbit	Amyloid beta (1–40)	2700791	1:100
Invitrogen	MN1050	Mouse	pTau Th181	YB3804861	1:500
Abcam	AB5076	Goat	Iba1	1069776-1	1:50
Abcam	AB4674	Chicken	GFAP	1088394-2	1:1000
Abcam	AB279296	Mouse	NeuN	1027822-16	1:200

### 2.5 High content slide scanning microscopy and deep learning-based image analysis

Stained slides were allowed to warm to RT under light protection before being loaded into an Olympus SLIDEVIEW™ VS200 scanning microscope (Evident, Waltham, MA, USA) equipped with a Hamamatsu ORCA-Fusion camera (C14440-20UP, Hamamatsu Photonics, Shizuoka, Japan). Images were collected using OlyVIA. Quantitative analysis of NeuN^+^ and Iba1^+^ cell counts, GFAP^+^ area, total amyloid-*β*^+^ area, and pTh181-Tau^+^ area were accomplished via machine learning using neural networks trained for approximately 100,000 iterations across all experimental groups (Visiopharm software, 2025.08.2). Networks were trained on 20X hippocampal montage images from 2 to 3 sections per animal (*n* = 4 to 6 animals/sex/group) collected using an Olympus UPLXAPO20X (NA 0.8) objective (0.274 μm/pixel). Pre-defined regions of interest (ROIs) were drawn for the CA1, CA2, CA3, and dentate gyrus hippocampal subregions using common anatomical landmarks referenced using a rat brain atlas.

### 2.6 Microglial morphometric analysis

Morphology of microglia was determined in Iba1 immunolabeled 40 μm tissue sections based on 40X Z-stack images (25 μm total depth, 1 μm step size) acquired using an Olympus VS200 scanning microscope and a UPLXAPO40X (NA 0.95) objective (0.137 μm/pixel). Images were imported into IMARIS for Neuroscientists software (version 10.2.0, Bitplane, South Windsor, CT, USA) and five to ten randomized planes of view were assessed for both plaque-associated and non-plaque-associated microglia per brain section. The Filament Tracing module in IMARIS was used to identify Iba1^+^ microglial processes originating from the soma of each detected cell via deep learning-based seed point and segment classification overseen by technicians blinded to experimental groups. The following parameters were assessed for each group: total sum of cellular area, total sum of branch lengths/cell, and number of branches/cell.

### 2.7 Statistical analysis

All data are presented as mean ± SEM, unless otherwise noted in the respective figure caption. Experimental values from each sex and experimental group were subjected to outlier analysis and exclusion via ROUT (*α* = 0.05). Statistical tests were determined by the number of variables being compared between groups via either two-way or three-way ANOVA with Bonferroni post-hoc correction. Statistical analyses were performed using GraphPad Prism (version 10.1.1; GraphPad Software, San Diego, CA, USA) unless otherwise stated in the above methods.

**Table 2:** Secondary antibody concentrations and associated product information.

Brand	Product #	Host	Antigen	Fluorophore	Lot #	Dilution
Invitrogen	A78948	Donkey	Chicken	AF488	2850198	1:500
Invitrogen	A31572	Donkey	Goat	AF555	2831376	1:500
Invitrogen	A31571	Donkey	Mouse	AF647	2720365	1:500
Invitrogen	SA5-10043	Donkey	Rabbit	DyLite 755	AB4626751	1:250

## 3 Results

### 3.1 DNA modification overview characteristics

**Figure 1:**
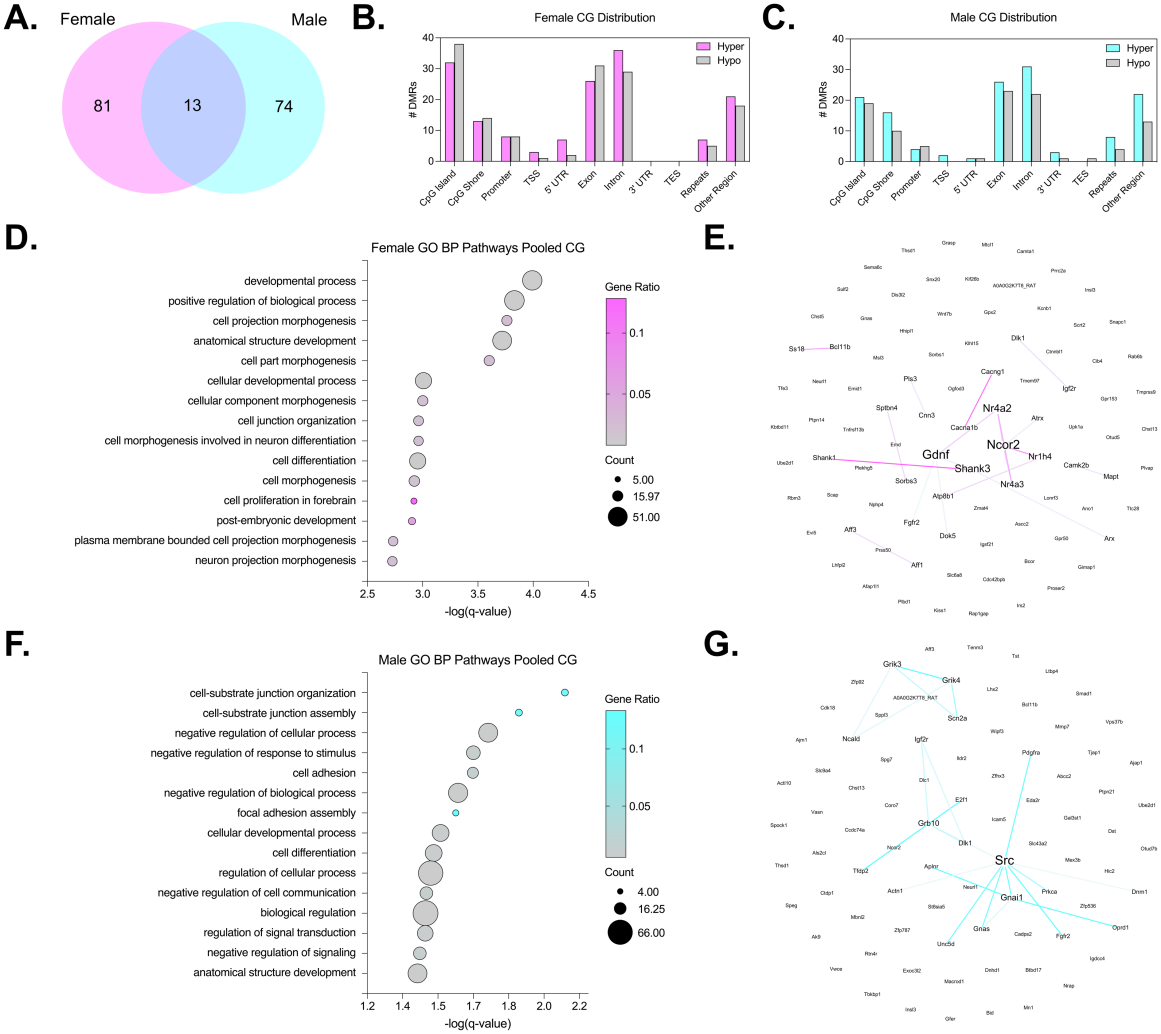
DNA modification patterns vary distinctly by sex and exercise status in aged transgenic rats. **(A)** Overlap assessment of the number of genes annotated from CG-associated DMRs by sex. **(B,C)** Number of CG, CHG, or CHH context DMRs across genomic feature by sex. Gray boxes represent hypomodified DMRs while colored boxes represent hypermodified DMRs. **(D,F)** Bubble plots depicting GO BP pathways enrichment analysis results by sex. X-axis represents -log(adj *p*-value), color scale depicts the ratio of genes associated with CG-context DMRs compared to the number of genes in each pathway, size of each bubble marker represents the number of DMR-associated genes associated each pathway. **(E,G)** PPI network maps visually representing known and predicted interactions between CG-context DMR annotated genes. Size of node correlates to number of edges, color scale correlates with strength of the interaction. *n* = 4 animals/sex/group for all epigenetic analyses.

**Figure 2:**
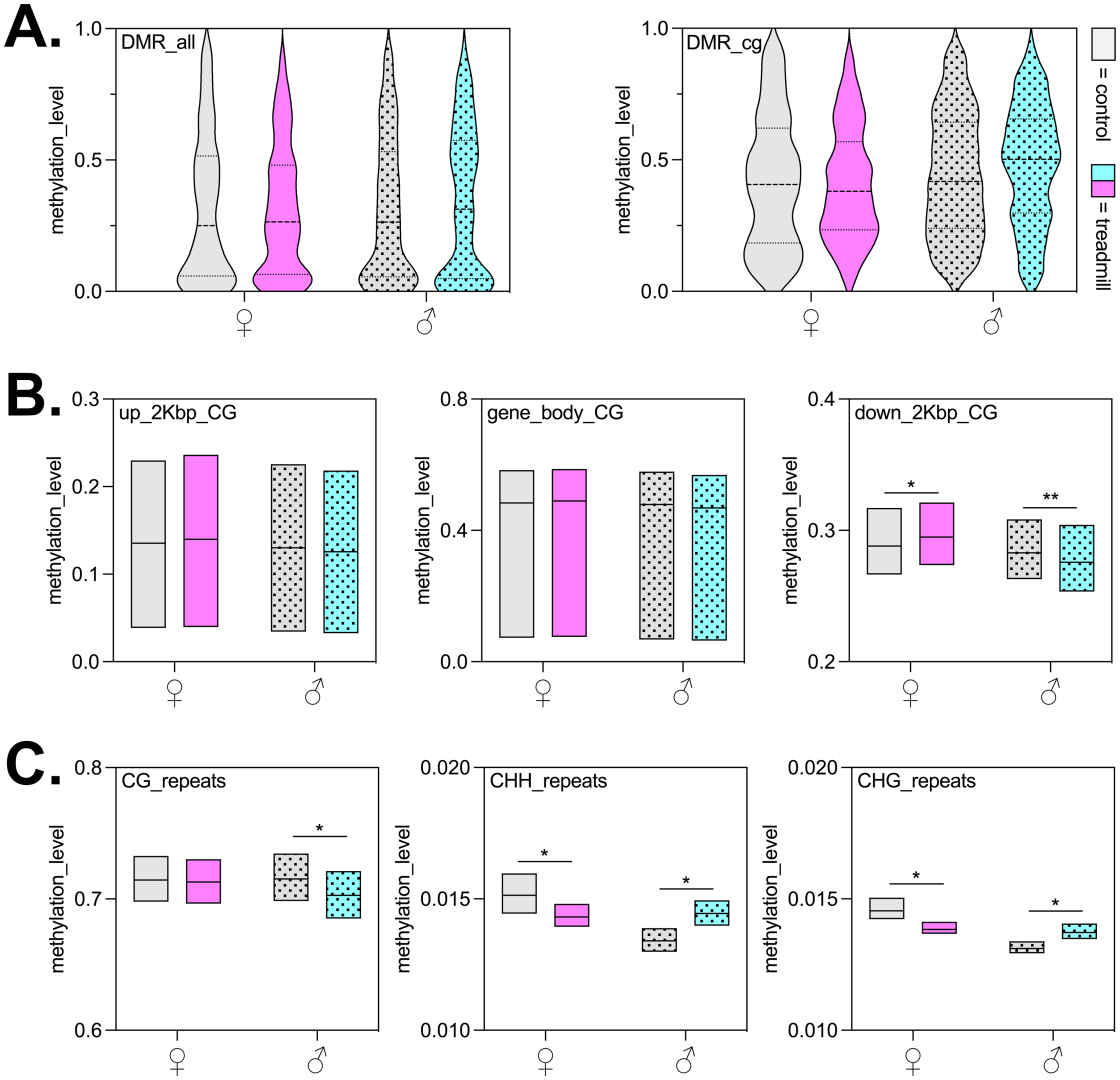
Epigenetic patterns are differentially modified in non-CG contexts relevant to Alzheimer’s disease. **(A)** Global DNA modification patterns for control or treadmill trained animals separated by sex and DMR status across all regions or in CpG-specific context. **(B)** DNA modification levels in upstream 2,000 bp from transcriptional start site, in the gene body, or downstreatm 2,000 bp in CG context, significant differences were identified via two-way ANOVA; *, ** denotes adjusted *p*-value < 0.05, 0.01, respectively. **(C)** CG, CHH, CHG repeat DNA modification levels separated by sex and exercise status; significant differences were identified via two-way ANOVA; *denotes adjusted *p*-value < 0.05. In all panels, magenta denotes treadmill trained females and cyan denotes treadmill trained males; *n* = 4 animals/sex/group for all epigenetic analyses.

To determine genome-wide epigenetic profiles in exercised animals compared to non-treadmill trained counterparts, approximately 33,500,000 total reads were captured via RRBS per sample (*n* = 4 animals/sex/group) with ~65% mapping rate (>20 million mapped reads per sample). Bisulfite conversion was greater than >99% for all samples. Global DNA modification patterns were assessed and DMRs were identified across the genome with 216 in males (148 CG context) and 239 in females (176 CG context). The distribution of these DMRs by associated genomic feature is shown in Figures 1B,C. Genome-wide analysis of all DMRs revealed a slight elevation in modification of CG-context DMRs following treadmill training in male animals (control median = 0.4177; treadmill median = 0.5019) which was inversely demonstrated in female animals (control median = 0.4059; treadmill median = 0.3801). This opposite directional change between sexes persisted when separating DMRs into 50 bp bins across promoter and gene body regions of the genome, CHH/CHG repetitive elements, and most significantly in the 2,000 bp downstream of the TES (Figure 2B). Additionally, changes in CG context repetitive element DNA modification levels were found to be significantly lower in treadmill trained male animals (mean difference = 0.01246; *p*-adj. = 0.0008) than in control males with no changes between females by exercise status (Figure 2C). The greatest number of DMRs in males were found to exist in exonic and intronic genome features with more hyper-modified regions than hypo-modified regions (Figure 1B). Female animals demonstrated the greatest number of DMRs in CpG islands with fewer hyper-modified regions than hypo-modified regions (Figure 1C).

### 3.2 DMR functional enrichment analysis

To assess the functional relevance of these epigenetic alterations across the genome, CG-context DMRs were annotated to protein-coding genes as described in the methods. 74 genes were uniquely mapped to male DMRs while 81 genes were only associated with female DMRs (Figure 1A). Additionally, 13 genes overlapped between sexes with 3 of those being concordantly disrupted DMRs: insulin-like factor 3 (*Insl3*), fibroblast growth factor receptor 2 (*Fgfr2*), and guanine nucleotide binding protein, alpha stimulating activity (*Gnas*). DMR-associated genes were further subjected to GO BP pathways overrepresentation analysis which highlighted terms relevant to barrier function and signal transduction in males (Figure 1F) and terms associated with neuroplasticity and cell morphologic change in females (Figure 1D). Both sexes did share one overlapping pathway: cell differentiation. DMR-associated genes were further characterized by utilizing the STRING database to construct protein–protein interaction (PPI) network maps. In male animals, this revealed a central hub surrounding SRC proto-oncogene, non-receptor tyrosine kinase (*Src*) (Figure 1E), while in females there existed two central nodes with a high degree, one around glial-derived neurotrophic factor (*Gdnf*) and the other encapsulating nuclear receptor co-repressor 2 (*Ncor2*), both of which associate with microglia and astrocytes (Figure 1G).

### 3.3 Quantification of NeuN^+^ cell density in the hippocampus

**Figure 3:**
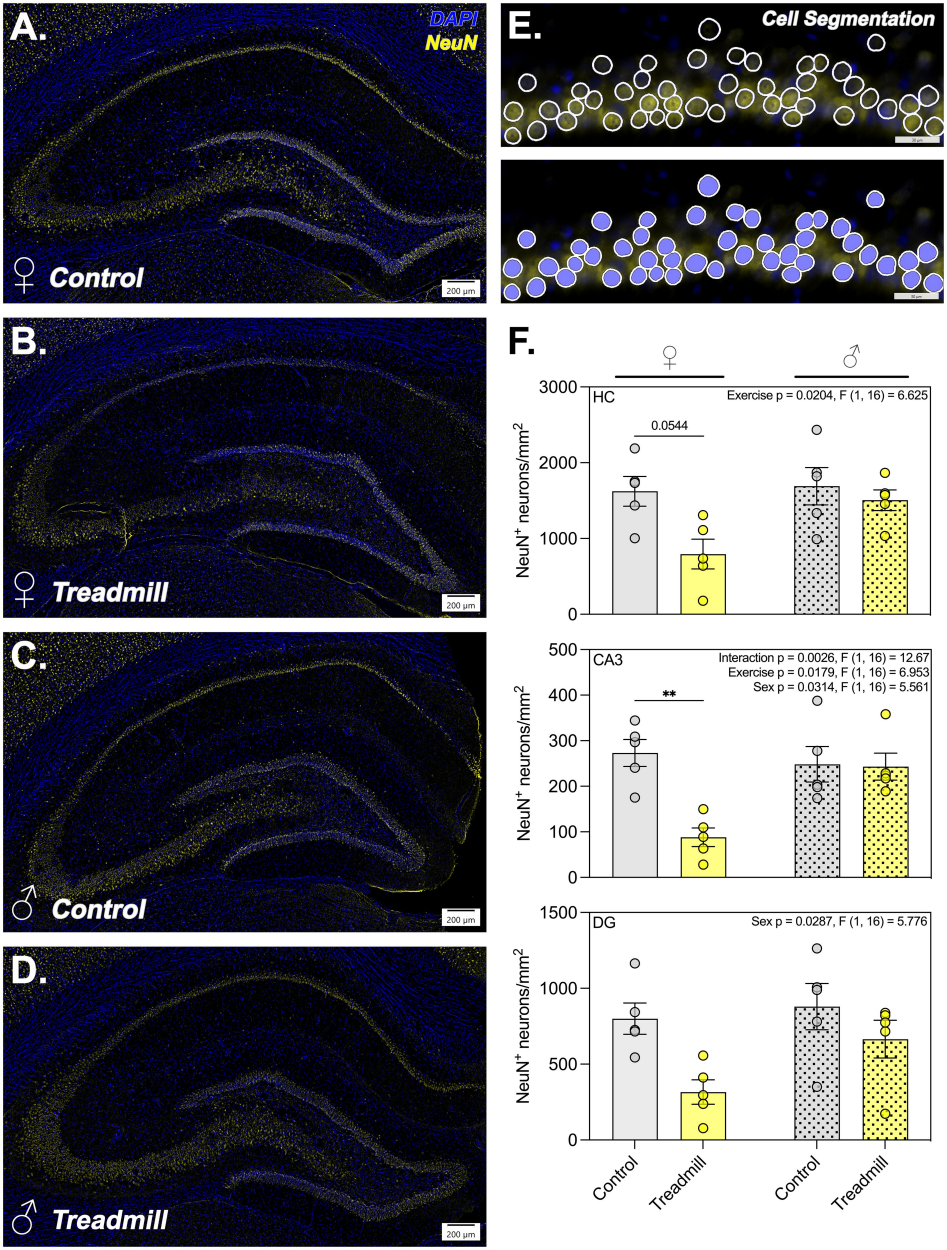
Hippocampal neuronal density is reduced following treadmill training in aged female but not male animals. **(A–D)** Representative hippocampal montage overviews depicting all analyzed subregions and associated staining of neuronal somas with NeuN (yellow) and nuclei with Hoechst 33342 (blue, labeled DAPI in panel). The bottom left hand corner of each image details the animal sex and exercise status. **(E)** Close-up image representing the efficiency of the cell segmentation neural network with unclosed and closed masks identifying each cell. **(F)** Bar graphs demonstrating the neuronal density calculated using Visiopharm across the hippocampal extent and by subregion for the CA3 and dentate gyrus ROIs. Each data point represents the average across 23 analyzed sections per animal. In the top right corner of each panel, main and/or interaction effects are depicted as identified using two-way ANOVA. *Denotes adjusted *p*-value < 0.05; *n* = 4–6 animals/sex/group for all image analysis.

To determine the effects of the treadmill training regimen on the density of neurons (NeuN^+^ cells/mm^2^) in the hippocampus, we employed high content imaging and deep learning-based image analysis to quantify the NeuN^+^ soma in each hippocampal subregion (Figures 3A–D). Unbiased neural networks were trained across the entire dataset to include both exercised and non-exercised animals and were assessed for accuracy after each 25,000 training iterations. When cellular detection/segmentation was deemed sufficient, as represented in Figure 3E, networks were batch applied to analyze all images. Female treadmill-trained animals exhibited significantly fewer NeuN^+^ cells/mm^2^ in the CA3 subregion than their non-exercised counterparts (mean difference = 185.1; *p* = 0.0028). Male animals had no statistically significant pairwise comparisons either by total hippocampal volume or by hippocampal subregion. These results indicated a significant main effect for sex across the entire HC (*p* = 0.0204; *F*[1,16] = 6.625) and in the CA3 region (*p* = 0.0287; *F*[1,16] = 5.776). In the DG, there were specific exercise- (*p* = 0.0314; *F*[1,16] = 5.561) and sex-dependent (*p* = 0.0179; *F*[1,16] = 6.953) main effects as well as an interaction effect (*p* = 0.0026; *F*[1,16] = 12.67).

### 3.4 Quantification of phosphorylated tau and amyloid protein burden in hippocampal subregions

**Figure 4:**
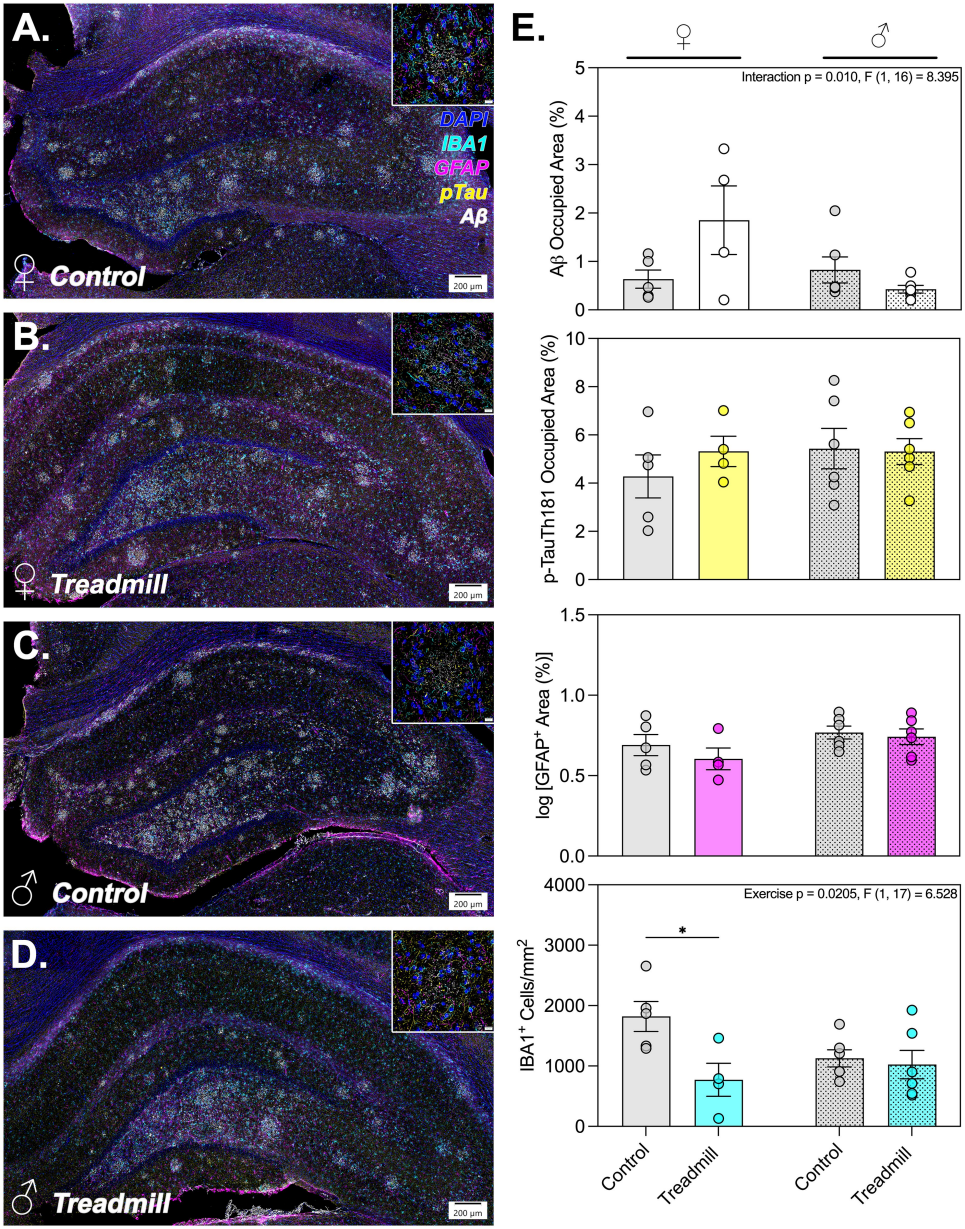
Hippocampal glial reactivity and aggregated pathologic protein burden vary by sex and exercise status. **(A–D)** Representative hippocampal montage overviews depicting all analyzed subregions and associated staining of microglia with Iba1 (cyan), astrocytes with GFAP (magenta), phosphorylated tau at Th181 (yellow), and amyloid-*β* 1–40 (white) as well as nuclei counterstained with Hoechst 33342 (blue, labeled DAPI in panel). The bottom left hand corner of each image details the animal sex and exercise status. Insets in the top right hand corner of each image demonstrate the reactivity of each marker by group. **(E)** Bar graphs demonstrating the tau and amyloid-β load, % GFAP+ area, and density of Iba1+ cells calculated using Visiopharm across the hippocampal extent. Each data point represents the average across 2–3 analyzed sections per animal. In the top right corner of each panel, main and/or interaction effects are depicted as identified using two-way ANOVA. *Denotes adjusted *p*-value < 0.05; *n* = 46 animals/sex/group for all image analysis.

To identify the effects of motorized exercise on the extent of amyloid protein burden in the hippocampus, we examined the occupied % area of the total HC and subregions using high content imaging and deep learning-based analysis to determine both total amyloid beta (Aβ 1–40) and phosphorylated tau (pTau-Th181). In multiplex immunolabeled images analyzed across the hippocampal extent, we observed a significant interaction effect for Aβ occupied % area across the entire extent of the HC (*p* = 0.0105; *F*[1,16] = 8.395) and in the CA1 subregion (*p* = 0.0168; *F*[1,16] = 7.131) as demonstrated in Figure 4E. In analyzing the HC as a whole, there was a sex-specific main effect (*p* = 0.0398; *F*[1,16] = 5.008). There were no main or interaction effects that varied significantly regarding pTau-Th181 occupied % area across the entire extent of the HC or by subregion (Figure 4E). There were also no statistically significant pairwise comparisons by exercise status for Aβ 1–40 or pTau-Th181 in either sex (Figure 4E).

### 3.5 Quantification of hippocampal microglia and astrocytes

To better understand the impact of glial activation on the observed sex-dependent differences in neuronal number and pathologic protein accumulation in the HC, we characterized the number of microglial cells and the extent of astrocytosis by quantitative immunofluorescence (Iba1^+^ cells/mm^2^ and % GFAP^+^ area, respectively). This revealed a significant exercise-dependent main effect for Iba1^+^ cells/mm^2^ in the whole HC (*p* = 0.0205; *F*[1,17] = 6.528) as shown in Figure 4E. Further, we observed a significant reduction in the number of Iba1^+^ cells/mm^2^ in treadmill trained females compared to non-exercised counterparts in the HC (mean difference = 104,762; *p* = 0.0418) and in the CA3 subregion (mean difference = 38,620; *p* = 0.0070) (Figure 4E; Supplementary Figure S2). No significant pairwise comparisons were observed for male animals regarding Iba1^+^ cells/mm^2^ or for either sex when examining GFAP^+^ area by whole HC or subregion (Figure 4E; Supplementary Figure S2).

### 3.6 Determination of microglial morphology

**Figure 5:**
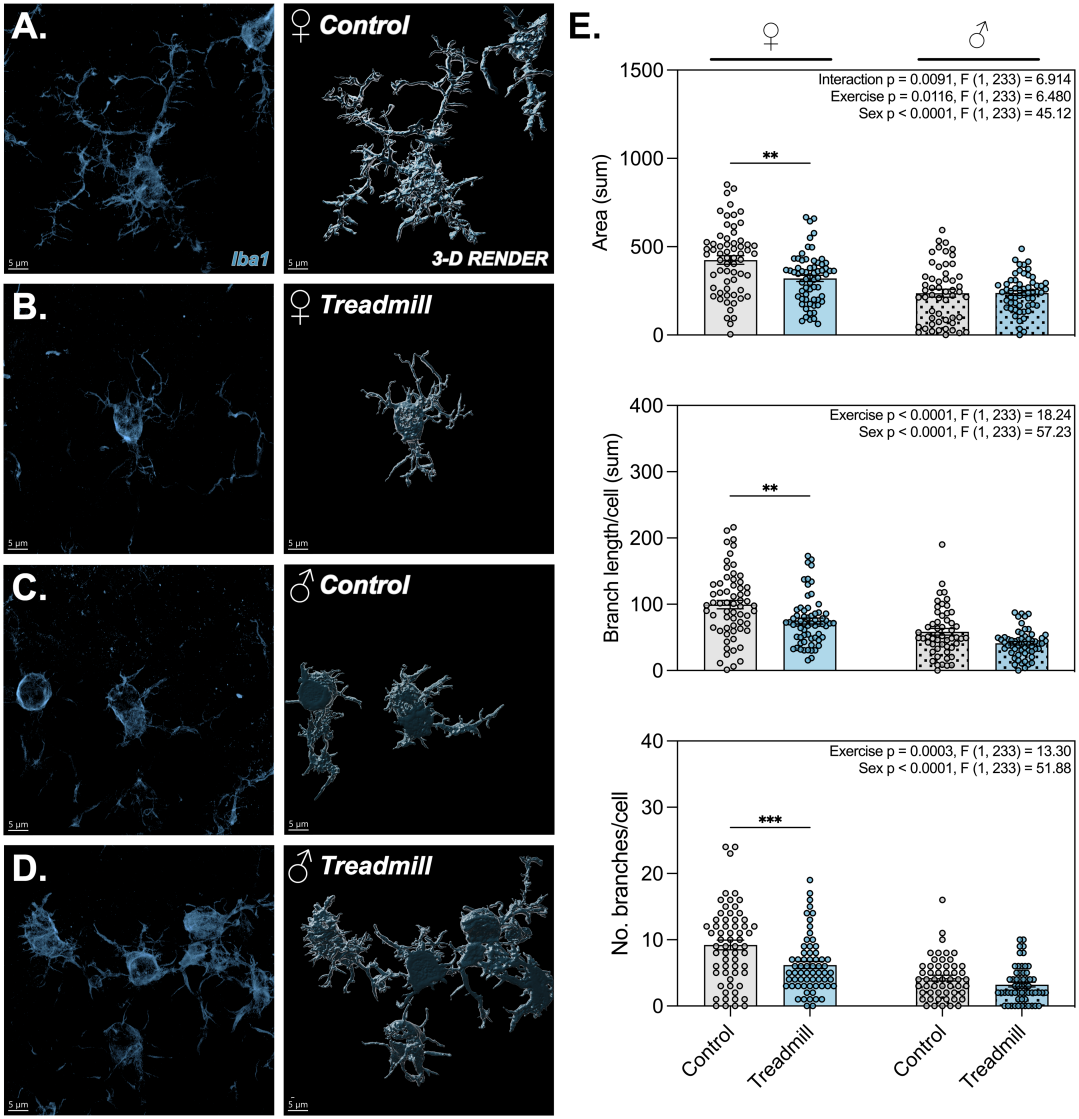
Microglia demonstrate significantly more ameoboid morphometric parameters in female treadmill trained animals compared to controls. **(A–D)** Representative high magnification images of plaque-associated microglia identified using Iba1 (muted blue). Next to each representative image is a respective 3-D render created using IMARIS. The top left hand corner of each 3-D render details the animal sex and exercise status. **(E)** Bar graphs demonstrating the total area, branch length/cell, and number of branches/cell calculated using IMARIS across 3–5 reference areas per animal. In the top right corner of each panel, main and/or interaction effects are depicted as identified using two-way ANOVA. **, ***Denotes adjusted *p*-value < 0.01, 0.001 respectively; *n* > 50 cells/sex/group across 4–6 animals/sex/group for all morphometric analysis.

Based on the changes observed in the density of microglia in the CA3 subregion of the hippocampus, we decided to further characterize the morphological phenotype of this cell population for both plaque-associated and non-plaque-associated microglia. To do this, we performed deep learning-based IMARIS filament tracing on Iba1^+^ cells across 3–5 randomly selected high magnification z-stack images per animal (>50 cells/group) of CA3 microglia. The application of three-dimensional morphometric analysis in a single cell manner has been evidenced as a highly sensitive technique used to identify important morphologic variation by treatment status in microglia compared to previous approaches (Murray et al., 2025). We observed female-specific alterations in the morphometric features of plaque-associated microglia in treadmill-trained animals compared to cage-confined controls, including a reduction in total cellular area, branch length/cell (sum), and number of branches/cell (Figures 5A–E). Interestingly, no differences were observed across non-plaque-associated microglia by sex or exercise status (Supplementary Figure S3).

## 4 Discussion

Physical exercise is widely regarded as beneficial for cognitive function and is commonly proposed as a potential lifestyle factor to mitigate worsening pathology associated with AD. However, under certain conditions or in specific populations, physical exertion itself may also contribute to physiologic stress, potentially contributing to underlying mechanisms of disease in individuals with AD. Here, we explored the effects of implementing a progressive treadmill training regimen in aged transgenic AD rats in order to examine sex-specific differences in histopathologic phenotype and DNA modification patterns in the hippocampus. We found robust, genome-wide differential cytosine modification profiles, both in CpG and non-CpG contexts, that varied by sex and exercise status. These epigenetic differences were accompanied by a female-specific decrease in total NeuN^+^ nuclei in the CA3 subregion, a significant decrease in Iba1^+^ cells across the entire hippocampus, and a sex-specific effect of exercise status on Aß load. Female plaque-associated microglia further demonstrated a more disease-associated morphologic phenotype after treadmill training, although this was not observed in non-plaque-associated microglia. These findings suggest that a more nuanced examination of the effects of exercise on AD and related disorders is warranted within the context of pre-existing disease status, age, and disease progression.

Epigenetic patterns, specifically DNA modification profiles, are increasingly recognized as distinct during disease states when compared to healthy controls (Zoghbi and Beaudet, 2016). In the aging brain, DNA methylation has also been shown to exist in hypo-modified states (Liu et al., 2009) when compared to younger individuals. Furthermore, broad disruption of epigenetic patterns has been reported in AD, both in the brain parenchyma (Klein and De Jager, 2016; Huo et al., 2019) and in peripheral tissues (Bonham et al., 2025; Sun et al., 2023). However, the directionality of these changes is not concordant between reports, with some studies reporting DNA hypermodification in disease and others reporting hypomodification in this context (Chouliaras et al., 2013; Bradley-Whitman and Lovell, 2013; Phipps et al., 2016; Madrid et al., 2018). Discrepancies between reports may be due to differences in the brain region examined, sex, cell type heterogeneity, and the technique used to characterize epigenetic profiles (Sanchez-Mut and Graff, 2015; Signal et al., 2023). Moreover, hippocampal epigenetic patterns are increasingly shown to contain alterations following physical exercise (Fernandes et al., 2017; Raus et al., 2023). Interestingly, there is a clear discordance in the epigenetic response to exercise in individuals who have a regular history of physical activity compared with those who are active only sporadically (Geiger et al., 2024). There are also reports of differential epigenetic responses based on the varying intensity of the exercise conducted, regardless of pre-existing physical fitness status (Hariharan et al., 2025).

In the present study we examined the DNA modification profile of the hippocampus in aged transgenic AD rats following a progressive treadmill training regimen through examination of both global DNA modifications as well as point-specific epimutations. In a whole-genome context, we report concordant hyper-modification globally when comparing CpG and non-CpG DMR-contexts following treadmill training in male animals (Figure 2A). Conversely, female rats demonstrated a CpG-specific decrease in modification status across DMRs (Figure 2A) which is consistent with the decreased global epigenetic patterning at CpG sites identified in multiple human cohorts of AD, including in monozygotic twins with AD discordance (Mastroeni et al., 2009), and in a randomized controlled trial of 20 AD patients vs. cognitively normal controls (Mastroeni et al., 2010). The latter study, and other more recently published reports (Shireby et al., 2022; Phipps et al., 2016), have also described cell-type specific changes in DNA modification patterns which were not detectable in our present study due to the tissue-wide epigenetic sequencing approach. Still, the DNA modification patterns explored here highlight changes in CpGs annotated to certain genes that have established roles in AD-relevant cell types. This includes increased modification of cytosines in the intronic and exonic regions of glial-derived neurotrophic factor (*Gdnf*) which was strongly connected to the DMR-annotated genes in the female PPI network map and displays a high degree of specificity for astrocytic release (Kuno et al., 2006). Functional enrichment analysis further revealed strong network interconnectedness surrounding nuclear receptor corepressor 2 (*Ncor2*) and nuclear receptor subfamily 4 group A member 2 (*Nr4a2*) in female treadmill trained animals. Both of these genes are known to play important roles in the regulation of microglial inflammatory signaling in AD (Yeh and Ikezu, 2019; Jakaria et al., 2019). The DMRs annotated to these genes were hypermodified in transcriptional regulatory regions, consistent with previous reports in peripheral tissues demonstrating heightened methylation and decreased transcript levels of these markers following prolonged exercise (Ronn et al., 2013). Lastly, SH3 and multiple ankyrin repeat domains (*Shank3*) was found to also possess substantially greater modified cytosines in female exercised animals than cage-confined controls. Decreased expression of this marker has been associated with synaptic loss and worsened outcomes in AD mice (Landry et al., 2023). It is worth noting that these shifts in DNA modification status warrant future validation with changes in functional gene expression. Still, these data motivated our subsequent characterization of neuronal and glial changes in this context.

Work examining the efficacy of motorized treadmill training in animal models to protect against AD-associated pathology reports mixed results (Belviranli and Okudan, 2019; Yuede et al., 2009). Consistent with this, histopathologic data in the present study suggest that progressive treadmill training during aging may exert sex-specific effects on hippocampal neurons in TgF344 rats, evidenced by a reduction in NeuN^+^ cell density in the CA3 in exercised females (Figures 3A,B,F). The CA3 subregion of the hippocampus is particularly sensitive to metabolic and excitotoxic stress due to its dense, recurrent circuitry and high glutamatergic activity, especially under pathological conditions such as Alzheimer’s disease (Zhang et al., 2013). Previous work investigating effects of intense physical exertion on neuronal viability in rats has also demonstrated sensitivity to neuronal apoptosis in this brain region (Ding et al., 2014) following a single bout of exhaustive swimming exercise. Additionally, evidence from a study examining long-term voluntary wheel running in rats suggests a potential for exercise-induced stress to limit hippocampal progenitor cell proliferation (Naylor et al., 2005). Reports of neuronal loss or impaired neuroplasticity following heightened physical exertion suggest that physiologic stress implemented during aging may exacerbate underlying vulnerability of hippocampal neurons. This may occur through mechanisms involving heightened oxidative stress or compromised mitochondrial energetics, both of which have been reported in the hippocampus of rats following certain exercise protocols (Sanguesa et al., 2022). Additional work remains to characterize the functional relevance of these histopathologic changes in cell density on behavioral outcomes relating to learning, memory, and executive function.

In addition to the changes in NeuN^+^ cell density, we report a significant decrease in the number of Iba1^+^ microglia across the entire hippocampus, and in the same CA3 subregion. Proliferation of microglia is commonly indicative of a disease-associated, pro-inflammatory state (Gomez-Nicola et al., 2013), whereas a decrease in microglial number is generally viewed as protective (Kohman et al., 2012; Kohman et al., 2013). Interestingly, despite the overall decrease in microglial number, morphologic analysis of plaque-associated microglia revealed a more amoeboid, disease-associated microglial (DAM) phenotype (Deczkowska et al., 2018), characterized by reduced total cell area, shorter process lengths, and decreased complexity of cytoplasmic arbors (Figure 5E). This shift suggests that prolonged physical exertion has the potential to impact hippocampal neuroinflammation by modulating microglial responses to Aß inclusions in older females. In healthy brains, microglia respond dynamically to environmental stimuli (Colonna and Butovsky, 2017), but under neurodegenerative conditions this activation may become maladaptive and promote synaptic pruning, cytokine release, or further neuronal injury (Wei and Li, 2022; Grubman et al., 2021; Valiukas et al., 2025). The increase in DAM-like morphologic phenotype aligns with previous work showing that environmental stressors, including forced exercise, can prime microglial cells toward a pro-inflammatory state (Inoue et al., 2015), especially in females who exhibit sex-specific immune responses (Osborne et al., 2018) as well as greater AD incidence. Why this change in reactive phenotype occurs specifically in plaque-associated cells, but non-plaque associated microglia remain unchanged, remains to be further explored as a future direction. Still, the shift toward DAM-like morphometric parameters in microglia proximal to Aß plaques aligns with the specific changes observed in pathologic protein accumulation in these rats, compared with largely unaffected tau tangles. It has been demonstrated that chronically activated microglia are less efficient at Aß endocytosis, suggesting that the reactive morphlogic state observed in the present study may contribute toward protein accumulation in this brain region (Sole-Domenech et al., 2016). More thorough characterization of glial cell dynamics throughout the time course of exercise implementation, and across progression of transgenic AD-like symptoms during aging, is needed to fully understand the subtleties of this complex relationship.

We acknowledge a few key limitations associated with the present study. One such feature is the inability to distinguish 5-methylcytosine marks from 5-hydroxymethylcytosine (5-hmC) marks. 5-hmC modifications are uniquely enriched in the brain parenchyma (Globisch et al., 2010; Stoyanova et al., 2021) and are thought to inversely affect gene expression canonically (Stoyanova et al., 2021), although the exact directionality of these mechanisms is not entirely understood. Follow up of this epigenetic profiling with emerging targeted techniques would aid in our ability to parse out the specific changes in epigenetic marks in this context. Additionally, a growing body of literature suggests that the neuronal marker NeuN is not homogenously expressed across certain neuronal populations, and that accordingly, decreased intensity of this cellular mark may not wholly indicate neuronal death (Moon et al., 2026; Guselnikova and Korzhevskiy, 2015). Thus, the changes in NeuN cell density observed in our present study cannot be definitively distinguished as transient responses to metabolic stress versus a true loss of cell number. Lastly, Iba1 is a marker of mature macrophages, including cells with resident origins in the periphery (Guselnikova et al., 2026). Accordingly, our present analysis of Iba1^+^ cell density is incapable of distinguishing between brain resident microglia and invading immune cells, which is a feature known to occur during aging and inflammation (Lier et al., 2021). Additional work is needed to understand the mechanisms underlying exercise-induced changes in these epigenetic and cellular phenotypes to increase our ability to apply this lifestyle modification in a therapeutic context.

Taken together, the data presented herein motivates additional work to determine if exercise is uniformly beneficial for brain health, particularly in the context of age-related neurodegeneration. While moderate physical activity has been widely shown to enhance synaptic plasticity and reduce neuroinflammation across the lifespan, our findings highlight a potential age and disease progression threshold beyond which physiologic stress may contribute to pathology under certain conditions. Furthermore, the observed female-specific effects may reflect differences in stress hormone responses, neuroimmune regulation, or mitochondrial resilience, all of which merit further investigation in this context. Our findings underscore the importance of exploring age of exercise onset, underlying disease status, and type of physical exertion as critical variables in the application of this lifestyle change as a therapeutic strategy for neurodegeneration. Future studies should aim to further delineate the mechanistic underpinnings of these effects and identify windows during the life course where physical activity would optimize neuroprotection without contributing to a parenchymal phenotype that might be less resilient to disease pathology, especially in vulnerable populations.

## Data Availability

The RRBS datasets analyzed in this study are publicly available in the Dryad Digital Repository at DOI: Dryad Digital Repository 10.5061/dryad.w0vt4b978. These data can be accessed without restriction and were used in accordance with the repository’s terms of use.
