# Supplementary material for: Treadmill training induces sex-dependent changes in hippocampal epigenetic patterns and plaque-associated microglial morphology in aged TgF344 rats
Source: Front Neurosci. 2026 May 5;20:1805957. doi: 10.3389/fnins.2026.1805957 (PMC13185687; doi:10.3389/fnins.2026.1805957)
Supplement: Supplementary file 2 [file Data_Sheet_1.DOCX]

Supplementary Material

# Supplementary Figures and Tables

## Supplementary Figures

##
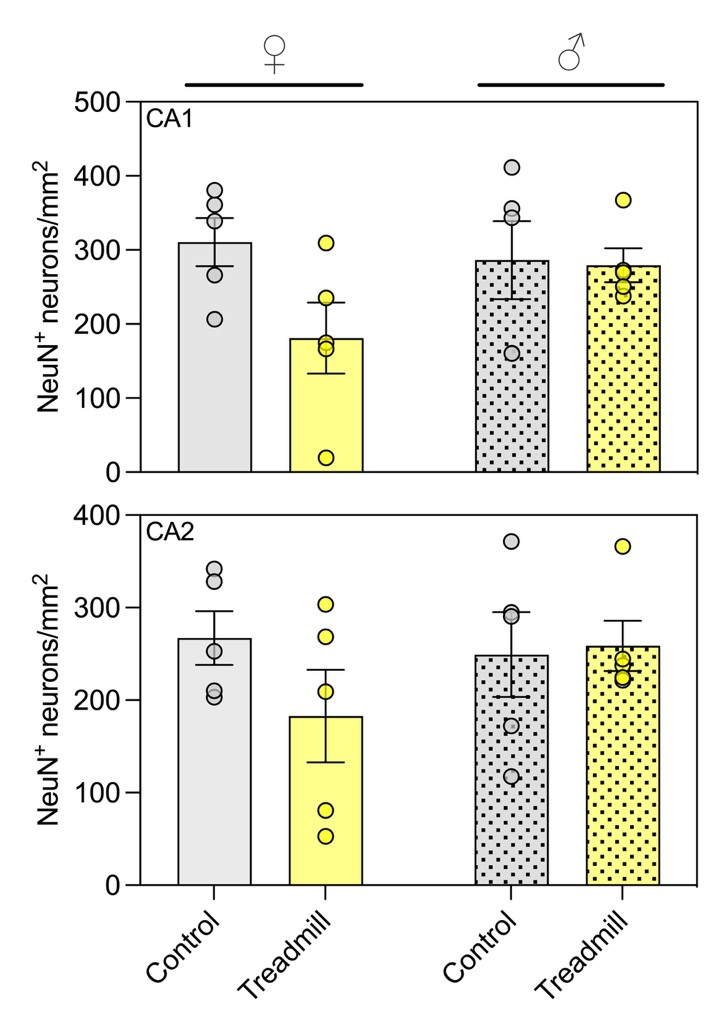
Supplementary Figure 1. CA1-CA2 neuronal density does not change following treadmill training in aged female or male animals. Bar graphs demonstrating the neuronal density calculated using Visiopharm by subregion for the CA1 and CA2 ROIs. Each data point represents the average across 2-3 analyzed sections per animal. Statistical differences were assessed using two-way ANOVA. n = 4-6 animals/sex/group for all image analysis.

**
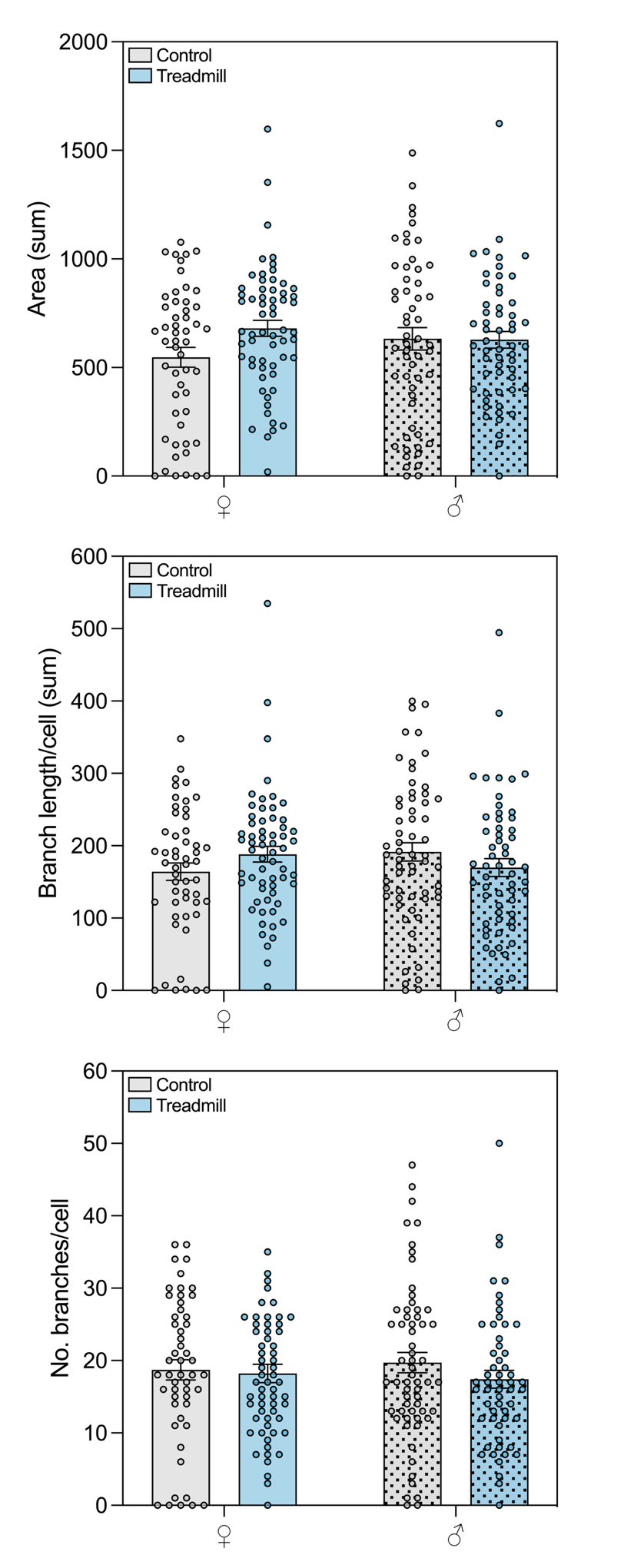
**

**Supplementary Figure 2.** **Non-plaque-associated microglial morphometric parameters do not vary by sex or exercise status.** Bar graphs demonstrating the area, branch length/cell, and no. of branches/cell determined via IMARIS. Each data point represents an individual cell. Statistical differences were assessed using two-way ANOVA. n = 4-6 animals/sex/group for all image analysis**.**

## Supplementary Tables

Attached in .excel format
